# How Clinical Integration of Pharmacists in General Practice has Impact on Medication Therapy Management: A Theory-oriented Evaluation

**DOI:** 10.5334/ijic.3291

**Published:** 2019-01-02

**Authors:** Ankie C. M. Hazen, Antoinette A. de Bont, Anne J. Leendertse, Dorien L. M. Zwart, Niek J. de Wit, Johan J. de Gier, Marcel L. Bouvy

**Affiliations:** 1 Department of General Practice, Julius Centre for Health Sciences and Primary Care, University Medical Centre Utrecht, Utrecht University, NL; 2 Institute of Health Policy and Management at the Erasmus University Rotterdam, NL; 3 Department of Pharmacotherapy, Epidemiology and -Economics, University of Groningen, NL; 4 Department of Pharmaceutical Sciences, Utrecht University, NL

**Keywords:** clinical pharmacist, integrated care, new roles, primary care, medication review

## Abstract

**Background:**

Data on medication-related hospital admissions suggest that there is an opportunity for improved pharmaceutical care. Hence, concerns about medication-related hospital admissions is a driver to extend and integrate the role of community pharmacists in general practice.

**Aim:**

The aim of this paper is to give a systematic description of 1) what integrating a non-dispensing pharmacist (NDP) in general practice entails and 2) how this integrated care model is expected to contribute to patients’ medication therapy management.

**Methods:**

Based on ethnographic data collected by NDPs in general practices in the Netherlands, we conducted a theory evaluation.

**Results:**

The impact of NDPs providing integrated care can be explained by 1) the specific expertise NDPs bring into general practice and the tailored solutions they offer for individual patients, including deviation from medical protocols when necessary, 2) the reconciliation of interprofessional tensions caused by overlapping tasks with practice nurses, which results in a distinct patient population, 3) the conduct of clinical medication reviews aligned to the work processes of the GP practice and 4) the integration of quality management work into clinical work.

**Conclusion:**

The success of integrated pharmaceutical care is dependent on how NDPs collaborate with GPs and practice nurses. NDPs need to mobilize clinical pharmaceutical expertise into general practice. Yet, integrating quality management into clinical work is key to integrate pharmaceutical care. Paradoxically, full integration requires from NDPs to develop a distinct role in general practice.

## Introduction

Data on medication-related unplanned admissions suggest that there is ample opportunity to improve pharmaceutical care. A systematic review reported that 7.1% of unplanned admissions were medication-related of which 59% were considered preventable [1]. The Dutch prospective multi-center study of Hospital Admissions Related to Medication (HARM) reported similar results with 5.6% of unplanned admissions being medication-related, of which nearly half were considered preventable [2]. The number of medication-related hospital admissions increases up to 10.4% within the aging population [3].

Pharmacists can play a vital role to address the problem of medication-related harm. In recent years, we have seen a profound change in the role of community pharmacists. Their role has shifted from compounding and dispensing medications to providing integrated pharmaceutical care [4,5]. The concept of pharmaceutical care emphasizes the pharmacists’ responsibility to pursue the best possible patient outcomes of medication therapy [6,7]. The implementation of pharmaceutical care may be hampered by traditional activities of pharmacists, related to the logistics and counseling of dispensing of medication. Embedding non-dispensing pharmacists (NDPs, also called clinical pharmacists, practice pharmacists) in general practice enables pharmacists not to be distracted from logistics but to primarily contribute to the quality of pharmaceutical care. Non-dispensing pharmacist are specialized health care professionals who perform patient-centered activities and services to develop and promote the rational and appropriate use of medication [8], and who are not involved in the dispensing of medication.

NDP-led services improve the quality of medications’ use and are increasingly implemented in the United States, Canada, Australia and the United Kingdom [9,10,11,12]. The key aspects of such an integrated care model are: being a team member, having access to medical records and performing medication therapy management. As a team member of a general practice, the NDP can easily contact the GP which fosters understanding and trust. Access to the medical records of patients supports pharmaceutical care provision and facilitates communication between NDPs and GPs [13,14,15]. Last but not least, the work of the NDPs is clinically focused, consisting of clinical medication reviews, consultations for medication related questions and targeted pharmaceutical care programs to systematically improve the quality of prescribing. It starts with individual or population-based problem identification and subsequently targets the problem in a patient-centered way.

**Figure 1:**
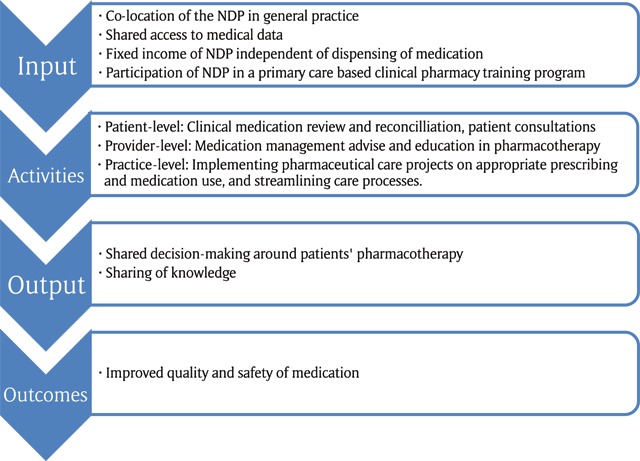
The preliminary program theory of clinical integration of NDPs in general practice.

The POINT practice model [16] incorporated key elements of the Canadian IMPACT model of NDP-care [17] (see ‘activities’, Figure 1) and assured alignment to the prevailing vision of pharmaceutical care provision [6,7]. In addition to the key elements we described in the former paragraph, we added two elements for the Dutch context, namely a fixed income and a training program. The fixed income made the NDP financially independent of dispensing of medication. The NDPs were trained by pharmacists and GPs in a primary care based clinical pharmacy training program [17] (See Input, Figure 1)

As the evidence for the benefits of this new role of pharmacists on real clinical endpoints such as mortality or medication-related hospitalisations is unknown [18], the POINT study group conducted a controlled intervention study, comparing clinical outcomes between NDP-led care and current models of pharmaceutical care delivery in the Netherlands. NDP-led care is new in the Netherlands

Measuring the clinical effectiveness of such a complex intervention is challenging given the variety of NDP-led services and different health care professionals involved. Hence, for optimal interpretation of the data, i.e. whether and how NDP-led care improves medication safety, we needed to describe the operational aspects of the intervention in detail [19]. The success of an integrated care model will be dependent on how the health care professionals collaborate, how work processes will be aligned and how tasks are reallocated. Therefore, in this particular study, we deliberately choose to focus on the process of clinical integration of the NDPs by observing their interactions with other health professionals in the primary care team. The aim of this paper was to give a systematic description of what is entailed in integrating an NDP as a member of the primary care team and how the integration could contribute to the safe and effective use of medication.

## Background

In the Netherlands, pharmaceutical care in primary care is currently provided by community pharmacists. The typical Dutch community pharmacy serves approximately 9,000 patients with one or two community pharmacists and eight pharmacy technicians delivering medication and health-related products. Dutch community pharmacists focus on counselling and dispensing of prescription medication. The majority of Over-The-Counter medication, food supplements and cosmetics are distributed through so called drugstores. In addition to dispensing fees, health care authorities have introduced a limited number of fees for pharmaceutical care services. In addition, community pharmacists can receive a higher dispensing fee if they score better on a selection of quality of care indicators.

General practice care in the Netherlands is increasingly organized in group practices. These group practices are at their turn increasingly located in community health centers together with community pharmacists, practice nurses and other health care providers. On average three to five general practitioners (GPs) with an extensive auxiliary staff provide primary care to 6,000–10,000 patients. GPs are paid by capitation (60%) and fee for service (40%), mainly consultations of ten minutes each. GPs receive bonus funding from insurance companies to meet the predefined quality of care indicators. Most GPs employ practice nurses specialized in chronic illnesses such as diabetes, cardiovascular disease and chronic obstructive pulmonary disease, and mental health staff addressing psychosocial problems. In addition to practice nurses, GPs employ practice assistants who do most of the triage, practice administration, and simple procedures.

## Methods

### Study design

We reconstructed the program theory of the integration of NDPs in general practice [20]. A program theory is a systematic description of what an intervention entails, how its elements link to the intended outcomes and how the intervention interacts with the context [19]. We distinguished in our preliminary program theory the input, the activities, the output and the outcomes. The input was: co-location of NDPs, shared medical records, a fixed income and participation of the NDP in a clinical pharmacy training program. The activities were: clinical medication reviews for patients with polypharmacy, medication reconciliation for patients discharged from the hospital, patient consultations about specific medication-therapy problems and targeted pharmaceutical care programs. The output was clinical integration in terms of knowledge sharing and shared decision making. The outcome was medication safety (See Figure 1).

### Setting and participants

Our unit of analysis were nine different NDPs. For a period of 15 months, these NDPs – all with previous work experience in community pharmacy – were posted in nine general practices [16]. Concurrently, the NDPs participated in an extensive general practice based Clinical Pharmacy Training Program to be prepared to work at the clinical side of primary care [17].

### Data collection

We trained the nine NDPs to do participative observations. These consisted of observing and describing any professional encounter during their work, e.g. conversations at lunchtime meetings on pharmaceutical care, the questions that GPs asked them during work, the reflections of the practice nurses and the practice assistants to their work and the role of managerial expertise in their work. The focus of the observations was defined upon both what is known about the introduction of new professional roles as well as upon the results of the first observations. Based upon what is known about new roles, we asked NDPs to observe how their work interacted with and possibly conflicted with the work of the GPs, the practice nurses and the practice assistants. Therefore, we asked the NDPs to observe the daily work of GPs, practice nurses and practice assistants. We asked them in particular to make notes about daily organization of the work in the practice and the interactions between practice nurses and GPs. To collect notes about their professional encounters during their work, we designed a data collection form in Excel. This form consisted of four headings: successes and challenges related to both task performance and aspects of clinical integration. The NDPs sent weekly updates of this form to the principal investigator (AH). In addition, we developed assignments that were incorporated in the educational program, related to clinical integration. An example of such an assignment was to compare and discuss task performance and responsibilities of practice nurses and NDPs. These completed assignments were sent to the principal investigator (AH).

Finally, we asked the NDPs to record how they spend their time by filling in an Excel-template representing five working days. Each day was divided in 15 minute timeslots. These standardized forms were filled in by the NDPs during three random weeks and sent to the principal investigator (AH) as well.

### Analysis

The researchers and the NDPs had monthly meetings in which they jointly analyzed the ethnographic data [21,22]. Although ethnographic research can refer to different methods of data collection and analysis, it can also be characterized by the – often lengthy – observation of interactions and behavior of participants in ‘real life settings’. Two researchers (AdB and AH) jointly performed a manual qualitative analysis of the notes on the encounters between the NDPs and the GPs or practice nurses. We identified commonalities and differences between the encounters. We studied nine NDPs and their collaborating GPs and we presented a selection of excerpts to the NDPs during the educational sessions of their clinical pharmacy training program. Subsequently, we asked the NDPs to reflect on the difference between successful and less successful encounters with regard to knowledge sharing and shared decision making. At the end of every lecture new instructions were provided for what the NDP needed to be noted down during their daily work in the clinic. For example, after the first round of observations the NDPs were asked to note down the questions GPs asked them about medication. We repeated this analytical process four times.

## Results

### Sharing expertise

We asked the NDPs to make notes of the questions GPs asked them. These questions provided insight in how knowledge was shared. In the first months of the study, the GPs asked the NDPs questions on medication. The questions were short and relatively uncomplicated and knowledge of the patient’s context was not essential. For example, a GP asked the NDP how to switch from the antidepressant citalopram to fluoxetine, since citalopram was not effective. In this case, the GP had already decided on the choice of medication therapy and only needed specific information about the best way to switch from one medication to the other. GPs appreciated concise and direct answers to these questions. The NDPs are supposed to mobilize pharmaceutical evidence on the spot.

After a couple of months, the GPs started sharing more complex cases with the NDPs. They appreciated pragmatic solutions for patients with more complex medication related problems. A GP asked for example if and how the NDP should stop citalopram for a patient with anxiety disorder. The NDP decided to invite the patient for counseling to discuss current medication-related needs, usage and experience with the medication (efficacy and side effects), concerns, potential complaints and the patient’s wish to stop the medication therapy. The NDP suggested a tailored scheme to stop citalopram and the NDP monitored the patient during the process of stopping. GP respected NDPs to go beyond protocols if necessary.

### Reconciliation of interprofessional tensions

Based upon the observations of the work of practice nurses, we analyzed how both the NDPs and the practice nurses reconciled interprofessional tensions over responsibilities and domain discussions. We reconstructed two strategies. The first strategy is to support the practice nurses in – what they call – difficult cases. NDPs take over the provision of pharmaceutical care to those patients who either did not fit well in the protocol or who used a medication that could be potentially dangerous. This strategy aimed to ease the work of the practice nurses. The following example was presented as a success by an NDP in her new role.

“The practice nurse referred a patient to the NDP. After three consultations, which resulted in adjusting antihypertensive medication, extra lab monitoring, stopping amitriptyline and starting vitamin B12, the patient was referred back to the practice nurse. I explained the medication changes to the practice nurse. The practice nurse said she appreciated our efficient collaboration and the insights into medication therapy. She added that she liked it that I (NDP) was so approachable.” (field notes of the NDP).

Another NDP explained:

“A practice nurse asked me that she would really appreciate extra training about medication and I am of course happy to provide that.” (field notes of the NDP).

The second strategy that we reconstructed was the identification of new patients. Based on specific observation of who initiated a patient consultation, we learned that the NDPs invited 69% of the patients for consultation. The GP initiated 13% of the consultations, other health care providers such as practice nurses initiated 7% and the patient initiated 11%. Hence, the NDPs do not seem to compete with practice nurses because the NDPs take up their own roles and identify their own distinct patient population. This is also reflected by the following:

“We regularly have interprofessional consultations with both the GP, practice nurse and me. During these meetings we discuss the patient’s treatment plan, make priorities and divide tasks. This works really well.” (field notes of the NDP)

### Clinical medication reviews

In the first months, the NDPs were instructed to focus their work of clinical medication reviews. They were asked to book a one-hour slot with patients for a clinical medication review. This hour would allow the NDP to study the whole patient record and especially a patient’s medication history. The GPs objected to a consultation of 60 minutes, and suggested a maximum of 20 minutes. Yet, the NDPs stressed that they needed more than an hour to discuss multiple problems, assess data, to contact other caregivers and make a sound clinical judgment. They compared their role with the role of a geriatric specialist, who can spend three hours on one patient. As an NDP explained:

“We try to unravel the puzzle, that takes time that is our strength. The GPs in my practice value the work that I do for patients.” (field notes of the NDP)

However, the importance to align with the work schedule of the GP practice is mentioned as well:

“On busy days, when a lot of patient consultations are scheduled, it is challenging to oversee all the patients. I am afraid to make mistakes. But I just need to learn to cope with this. This is how it works in general practice.” (field notes of the NDP)

By gaining more experience, the time of the intake consultation diminished, as noted by one of the NDPs:

“Today, the intake consultation only took 35 minutes. I am satisfied about the flow of the consultation and how we defined and prioritized the patient’s problems. In my experience, the consultation was very efficient. An intake of less than 30 minutes feels to be impossible though.” (field notes of the NDP)

Over time the clinical medication review developed in series of meetings. A clinical medication review consists on average of an intake consultation of 30 to 60 minutes in the patient’s home or in the general practice, followed by two to three short follow-up meetings in the patient’s home, by telephone or in the general practice.

### Clinical quality management

We asked the NDPs to introduce medication therapy quality management into their practice. As community pharmacists, the NDPs were already trained in quality management. We asked the NDPs to observe which clinical skills were relevant for quality management in general practices. One particular skill became prominent in the analysis of the different quality projects that the NDPs started. This skill was to invite patients to the clinic for medication therapy management.

“It is not easy to ask a patient to come to the clinic for a medication review. Some patients are not inclined to come. They are content with the medication they use.” (field notes of the NDP).

Patients tended not to come to the practice when they were invited to change or discontinue their medication therapy. Another NDP explained:

“I took the lead in implementing a quality project about the overtreatment with proton pump inhibitors. Instead of a list with eligible patients – that will often just pile up on the desk of the GP – I consulted almost all of the patients and evaluated their medication use.” (field notes of the NDP).

For a successful conduct of a quality project, it was necessary to thoroughly screen for eligible patients. An NDP stated:

“I do not particularly like the paperwork that comes with implementing a quality improvement project. The screening of eligible patients is very time consuming. But I realize that part needs to be done to perform clinical patient consultations.” (field notes of the NDP)

Rather than discussing whether medication should be stopped or changed, the NDP needed to start the consultation by discussing symptoms that might bother patients and then assess their needs regarding these symptoms. A good example was a quality management project that all NDPs performed on the use of alpha-blockers for lower urinary tract symptoms. The NDPs selected patients from the general practice who were prescribed medication for lower urinary tract symptoms and invited them to evaluate their medication use. By discussing symptoms and the effect of medication therapy, the NDPs experienced that the patients were more likely to discuss their medication-related needs, identify medication related problems and actually change their medication use.

## Discussion

As the success of integrated pharmaceutical care is dependent on how NDPs collaborate with GPs and practice nurses, how their work processes are aligned and thus how tasks are reallocated, we studied how NDPs perceive their interactions with GPs and practice nurses. Based upon observation notes made by the NDPs in their daily work at the clinic, we showed that NDPs need to bring first and for all clinical pharmaceutical expertise into general practice. Moreover, integrating quality management into clinical work is key to successfully align work processes and is the basis for task allocation. Paradoxically, full integration requires from NDPs to develop a distinct role in general practice.

Based upon the comparison of interactions with GPs and practice nurses that were perceived as successful, we showed that NDPs can share knowledge effectively in two distinct ways. First, NDPs need to mobilize pharmaceutical evidence on the spot. To collaborate successfully with GPs they need to provide direct answers to questions with regard to medication. Second, NDPs need to offer tailored solutions for problems of individual patients. Thereby, NDPs need to show that they can deviate from protocols if necessary *and* provide additional checks that allow deviation from the protocols.

Based upon the comparison of interactions with GPs and practice nurses over time, we showed that NDPs reconcile interprofessional tensions on time and tasks by framing their work as quality management. Hence, the integration of NDPs in general practice depends on their experience in quality management and their ability to apply this in the clinical setting of the general practice [23]. NDPs integrate managerial skills and clinical skills in medication therapy management and in quality improvement projects for patient at risk. Consequently, they provide care to a distinct patient population that is elderly patients with polypharmacy and multi-morbidities [23].

Although this was not the aim of this study, we noticed remarkably little resistance and tensions about role and responsibilities between community pharmacists and NDPs. Their roles can be complementary. Community pharmacists focus on dispensing medication, providing patient information and basic medication therapy management with incidental patient follow-up. NDPs focus on complex medication therapy management and patient consultations with structured follow-up.

This study adds to the literature on training in clinical pharmacy. Training in North-America, Australia, New Zeeland and the United Kingdom [9,12,18,24,25,26,27,28]. showed considerable variety. This study shows the importance of training clinical pharmacists to make evidence-based decisions in which they combine their knowledge of medication with their clinical experience, taking into account the context and the needs of the patient [29]. We assume that training in clinical reasoning and consultation skills [30,31] are key to shared decision making. The conceptual distinction between evidence and expertise is hereby relevant [32]. Evidence refers to the facts that can be transferred from one domain to the other – such as the active mechanism of a pharmaceutical, its benefits for a defined patient population and its possible side effects. Expertise is the professional ability to make judgments within a specific context [33], such as the decision to deviate from a prescription guideline or to suggest a non-pharmaceutical solution [34].

Previous research highlighted the challenge of overlapping roles between pharmacists and other health professionals, such as practice nurses, which could lead to conflicts [35]. This study shows that NDPs will not substitute care presently provided by GPs or practice nurses as they develop a distinct role. First, NDPs who integrate clinical pharmaceutical quality management into their role provide a complementary skill set to GPs and practice nurses. Second, they provide care to a distinct patient population, namely patients with multiple conditions who are at risk of medication-related problems. In fact, the added value of the integration on NDPs depends on the patient populations they identify. Previous research showed convincing evidence on pharmacist-led services for patients with a specific condition or specific medication [9,18,36]. Yet, the literature also showed that patients at risk of medication-related problems often have multiple conditions and polypharmacy and require a comprehensive medication therapy management approach. Given the considerable variety in the specific services that NDPs performed and their degree of (clinical) integration [9,12,18,24,25,26,27,28], this study underlines the need to address the distinct role of NDPs in general practice. The expertise of pharmacists can be better used when they have full responsibility for pharmaceutical care provision [37].

This study has limitations. First, the data in this study was collected by the NDPs and not by experienced researchers. Hence, the observation notes were less detailed. In addition, the focus of the study is limited to the perception of NDPs. How GPs and practice nurses perceived the interactions with NDPs is not addressed in this study. Similarly, the perception of patients is not taken into account. Studies on the effect of this integrated care mode on patient-related outcome measures are needed.

## Conclusion

The success of integrated pharmaceutical care is dependent on how NDPs collaborate with GPs and practice nurses. NDPs need to mobilize clinical pharmaceutical expertise into general practice. Yet, integrating quality management into clinical work is key to integrate pharmaceutical care. Paradoxically, full integration requires from NDPs to develop a distinct role in general practice.
